# Tick-borne Apicomplexa in wildlife and ticks of French Guiana[Fn FN1]

**DOI:** 10.1051/parasite/2024052

**Published:** 2024-08-15

**Authors:** Rachid Koual, Benoit de Thoisy, Xavier Baudrimont, Stéphane Garnier, Frédéric Delsuc, Olivier Duron

**Affiliations:** 1 MIVEGEC, University of Montpellier, Centre National de la Recherche Scientifique (CNRS), Institut de Recherche pour le Développement (IRD) 34394 Montpellier France; 2 Laboratoire des Interactions Virus-Hôtes, Institut Pasteur de Guyane 97300 Cayenne France; 3 Association Kwata ‘Study and Conservation of Guianan Wildlife’ 97300 Cayenne France; 4 Direction Générale des Territoires et de la Mer (DGTM) – Direction de l’environnement, de l’agriculture, de l’alimentation et de la forêt (DEAAF) 97300 Cayenne France; 5 Biogéosciences, University of Bourgogne, Centre National de la Recherche Scientifique (CNRS, UMR 6282) 21000 Dijon France; 6 ISEM, University of Montpellier, Centre National de la Recherche Scientifique (CNRS), Institut de Recherche pour le Développement (IRD) 34095 Montpellier France

**Keywords:** Ticks, Babesia, Theileria, Hemolivia, Hepatozoon, French Guiana

## Abstract

Tick-borne Apicomplexa encompass a group of parasites responsible for significant medical and veterinary diseases, including babesiosis, theileriosis, and hepatozoonosis. In this study, we investigated the presence and diversity of tick-borne Apicomplexa in wildlife and ticks inhabiting the Amazon rainforests of French Guiana. To this end, we conducted molecular screening and typing using 18S rRNA sequences on a collection of 1161 specimens belonging to 71 species, including 44 species of wild mammals, five species of passerines, and 22 species of ticks. We characterized eight genovariants of *Babesia*, *Theileria*, *Hemolivia,* and *Hepatozoon* parasites, some matching known species, while others suggested potential novel species. These parasites were detected in wild mammals, including opossums, sloths, armadillos, porcupines, margays, greater grisons, and ticks, but not in passerines. Finally, similarities with surveys conducted in Brazil highlight the specific sylvatic transmission cycles of South American tick-borne Apicomplexa.

## Abstract (fr)

Les Apicomplexes transmis par les tiques englobent un groupe de parasites responsables de maladies médicales et vétérinaires importantes, notamment la babésiose, la theilériose et l’hépatozoonose. Dans cette étude, nous avons étudié la présence et la diversité des Apicomplexes transmis par les tiques dans la faune sauvage et les tiques habitant les forêts tropicales amazoniennes de Guyane française. À cette fin, nous avons effectué un criblage moléculaire et un typage à l’aide de séquences d’ARNr 18S sur une collection de 1 161 spécimens appartenant à 71 espèces, dont 44 espèces de mammifères sauvages, cinq espèces de passereaux et 22 espèces de tiques. Nous avons caractérisé huit génovariants des parasites *Babesia*, *Theileria*, *Hemolivia* et *Hepatozoon*, certains correspondant à des espèces connues tandis que d’autres suggéraient de nouvelles espèces potentielles. Ces parasites ont été détectés chez des mammifères sauvages, dont des opossums, des paresseux, des tatous, des porcs-épics, des margays, des grisons et des tiques, mais pas chez des passereaux. Enfin, des similitudes avec des enquêtes menées au Brésil mettent en évidence les cycles de transmission sylvatiques spécifiques des Apicomplexa transmis par les tiques d’Amérique du Sud.

## Introduction

Tick-borne Apicomplexa are responsible for medically and veterinary relevant diseases, such as babesiosis, theileriosis (referred to collectively as piroplasmosis) and hepatozoonosis [17, 22, 23, 38, 43]. Members of the phylum Apicomplexa are unicellular eukaryotic organisms, with the majority of species being obligate endoparasites of animals, and in certain instances, of humans. Tick-borne Apicomplexa belong to two distinct orders: Piroplasmida, which includes the genera *Babesia* (M’Fadyean & Stockman, 1911), *Theileria* Bettencourt, França & Borges, 1907, and *Cytauxzoon* Neitz and Thomas, 1948 (piroplasmids), and Eucoccidiorida, which includes the genera *Hemolivia* Petit et al*.*, 1990 and *Hepatozoon* Miller, 1908 (haemogregarines) [17, 22, 23, 28, 38, 43, 49]. Piroplasmids are primarily transmitted through the bite of infected ticks, but the transmission of *Hemolivia* and *Hepatozoon* rather occurs through the ingestion of infected ticks and the release of the parasites into the host’s digestive system [17, 22, 23, 37, 38, 43]. Once inside the host, piroplasmids and haemogregarines mainly invade either red or white blood cells, but *Hepatozoon*, unable to replicate in these cells, further infects target organs where it causes disease [17, 22, 23, 37, 38, 43]. Despite representing a significant veterinary threat worldwide, and posing an emerging risk to humans, these blood parasites have received comparatively less attention than other vector-borne Apicomplexa, such as *Plasmodium*, their malaria-causing relative [17, 22, 23, 43].

Tick-borne Apicomplexa are the predominant group of mammalian blood parasites [17, 22, 23, 38, 43]. In domestic animals, various species elicit a spectrum of clinical signs, leading to substantial morbidity and mortality, often resulting in significant economic burdens. In cattle, representative species include *Babesia divergens*, *Babesia bovis*, *Babesia bigemina*, *Theileria annulata*, and *Theileria parva*. In dogs, *Babesia canis*, *Babesia gibsoni*, and *Babesia vogeli* are among the prominent species [17, 22, 23], while *Cytauxzoon felis* and *Hepatozoon felis* are significant in cats [23, 38, 43]. Wildlife also suffers from a wide array of tick-borne Apicomplexan species, affecting mammals, birds, reptiles, and amphibians with significant morbidity and mortality [17, 22, 23, 37, 38, 43]. Humans are not the natural hosts for tick-borne Apicomplexa, but babesiosis is increasingly recognized as an emerging zoonosis, with numerous critical clinical cases documented worldwide. Indeed, *Babesia microti*, typically found in mice and other small rodents, and *B. divergens*, mostly found in cattle, are the most common causes of human babesiosis, in North America and Europe, respectively [24, 27, 45–47].

Tick-borne Apicomplexa are classified and taxonomically identified based on their morphological, biological, and molecular characteristics [22, 23, 38, 43]. Molecular phylogenies using the 18S rRNA gene sequences have recently challenged the previous taxonomic classification of piroplasmids, revealing that *Babesia* and *Theileria* each represent polyphyletic groupings within their respective orders [23]. Hence, different *Babesia* species cluster with some *Theileria* species rather than forming cohesive monophyletic genera. However, phylogenetic approaches also indicate that each lineage of tick-borne Apicomplexa is typically associated with a restricted range of vertebrate host taxa, suggesting that these lineages have often evolved specializations to their respective vertebrate hosts [22, 23, 38, 43]. For instance, *Babesia* species of the Percei lineage exclusively infect birds, while species of the *Cytauxzoon* lineage are predominantly found in felids [23]. Members of the lineage *Babesia* sensu stricto form a notable exception, as they infect multiple and diverse mammalian and avian vertebrate hosts [23]. Nevertheless, surveys of wildlife continue to uncover new tick-borne Apicomplexa that do not fit into recognized species [4, 5, 13, 20, 21, 26, 30, 31, 34, 36, 43]. Their host range, and phenotypic and developmental characteristics remain unknown in many cases [23, 47].

In this study, we investigate the presence and diversity of tick-borne Apicomplexa in wild mammals, birds and ticks inhabiting the Amazon rainforests of French Guiana. This territory of 84,000 km^2^ is predominantly covered by old-growth rainforests hosting one of the highest biodiversities in the world [42]. While recent surveys have uncovered novel tick-borne microbes in French Guiana [7, 11, 12, 25, 32], including novel tick-borne pathogens infecting humans [14, 19], there have been few studies focusing on tick-borne Apicomplexa in this region. In 1988, *Babesia choloepi* was described as a novel species in Linnaeus’s two-toed sloths (*Choloepus didactylus*) of French Guiana based on morphological features [18], but no molecular sequences are available to complete its description. Furthermore, a survey of blood smears revealed the presence of several undetermined piroplasmid and *Hepatozoon* species in most mammalian orders of French Guiana, except primates, and in several species of snakes [41]. However, none of these previous studies have characterized these tick-borne Apicomplexa based on molecular typing.

## Materials and methods

### Ethics

Mammal samples were collected and used in accordance with an international CITES permit (Convention on International Trade in Endangered Species of Wild Fauna and Flora; permit FR973A) following French legislation. Following sharing policies in French Guiana, mammal samples are registered in the JAGUARS collection (https://kwata.net/gestion-collection-biologique; CITES reference: FR973A) supported by Kwata NGO (accredited by the French Ministry of the Environment and the Prefecture of French Guiana, Agreement R03-2019-06-19-13), Institut Pasteur de la Guyane, Direction Générale des Territoires et de la Mer (DGTM), Collectivité Territoriale de la Guyane, and validated by the French Guianese prefectural decree No. 2012/110. The French Ministry of Higher Education and Research provides authorization for projects using wild animals for scientific purposes (reference APAFIS-37571-2022111610578451). Permits for bird sampling (French Guiana prefectural decrees Nos. 2011/003, 2013/127 and R03-2018-10-30-0092) authorized the capture, marking, sampling, holding and transport of bird samples. Bird sampling was also done with permissions from several organizations: the DGTM de Guyane, the Direction Régionale de l’Office National des Forêts (ONF) de Guyane, the Conservatoire du Littoral, the Centre National d’Études Spatiales (CNES), the Centre Spatial Guyanais (CSG), the Association pour la Découverte de la Nature en Guyane, the association Randoroura. The use of bird genetic resources is declared to the French Ministry of the Environment under reference TREL1820249A/49 in accordance with the Nagoya Protocol on Access and Benefit Sharing (ABS). The French Ministry of the Environment also validated the collection and use of tick samples under the reference TREL19028117S/156, in accordance with the ABS Nagoya Protocol. All animals were handled in strict accordance with good animal practice and ethical standards as defined by the French code of practice for the care and use of animals for scientific purposes, established by articles R214-87 to R214-137 of the French rural code.

### Collection of samples

We used archived DNA templates extracted from 1161 specimens collected in French Guiana between 1994 and 2019 (Tables 1 and S1). This collection comprises samples from 71 species, including 44 species of wild mammals (*n* = 626 samples), five species of passerines (*n* = 247), and 22 species of ticks (*n* = 288). It includes blood samples (for wild mammals, and passerines), spleen samples (wild mammals), or whole body (ticks). We had primarily collected these samples as part of previous studies among wildlife, and ticks in French Guiana [7–10, 12, 14].

**Table 1:** List of mammal, passerine and tick species examined for the presence of tick-borne Apicomplexa in French Guiana. A detailed list of specimens and sampling locations is provided in Table S1.

Host species	Species local name	Order	Number of examined specimens	Number of infected specimens				
				*Babesia*	*Theileria*	*Cytauxzoon*	*Hepatozoon*	*Hemolivia*
**Mammals**								
*Caluromys philander*	Bare-tailed woolly opossum	Didelphimorphia	5	–	–	–	–	–
*Didelphis marsupialis*	Common opossum	Didelphimorphia	51	–	–	–	7	–
*Marmosa lepida*	Rufous mouse opossum	Didelphimorphia	1	–	–	–	–	–
*Marmosa murina*	Linnaeus’s mouse opossum	Didelphimorphia	20	–	–	–	–	–
*Marmosops parvidens*	Delicate slender opossum	Didelphimorphia	5	–	–	–	–	–
*Metachirus nudicaudatus*	Brown four-eyed opossum	Didelphimorphia	5	–	–	–	–	–
*Micoureus demerarae*	Woolly mouse opossum	Didelphimorphia	16	–	–	–	–	–
*Philander opossum*	Gray four-eyed opossum	Didelphimorphia	20	–	–	–	1	–
*Bradypus tridactylus*	Pale-throated three-toed sloth	Pilosa	108	–	–	–	–	–
*Choloepus didactylus*	Linnaeus’s wo-toed sloth	Pilosa	90	7	–	–	–	–
*Cyclopes didactylus*	Pygmy anteater	Pilosa	1	–	–	–	–	–
*Tamandua tetradactyla*	Southern tamandua	Pilosa	3	–	–	–	–	–
*Cabassous unicinctus*	Southern naked-tailed armadillo	Cingulata	2	–	–	–	–	–
*Dasypus novemcinctus*	Nine-banded armadillo	Cingulata	15	–	3	–	–	–
*Hydrochoerus hydrochaeris*	Capybara	Rodentia	2	–	–	–	–	–
*Holochilus sciureus*	Amazonian marsh rat	Rodentia	5	–	–	–	–	–
*Hylaeamys megacephalus*	Large-headed rice rat	Rodentia	15	–	–	–	–	–
*Hylaeamys yunganus*	Yungas rice rat	Rodentia	10	–	–	–	–	–
*Neacomys dubosti*	Dubost’s bristly mouse	Rodentia	1	–	–	–	–	–
*Neacomys paracou*	Paracou bristly mouse	Rodentia	8	–	–	–	–	–
*Nectomys rattus*	Small-footed bristly mouse	Rodentia	4	–	–	–	–	–
*Oecomys auyantepui*	North Amazonian arboreal rice rat	Rodentia	16	–	–	–	–	–
*Oecomys bicolor*	Bicolored arboreal rice rat	Rodentia	16	–	–	–	–	–
*Oligoryzomys fulvescens*	Fulvous pygmy rice rat	Rodentia	7	–	–	–	–	–
*Makalata didelphoides*	Brazilian spiny tree rat	Rodentia	8	–	–	–	–	–
*Mesomys hispidus*	Ferreira’s spiny tree-rat	Rodentia	13	–	–	–	–	–
*Proechimys cuvieri*	Cuvier’s spiny-rat	Rodentia	18	–	–	–	–	–
*Proechimys guyannensis*	Guyenne spiny-rat	Rodentia	20	–	–	–	–	–
*Coendou melanurus*	Black-tailed hairy dwarf porcupine	Rodentia	1	–	–	–	–	–
*Coendou sp.*	Prehensile-tailed porcupines	Rodentia	3	–	–	–	1	–
*Mus musculus*	House mouse	Rodentia	34	–	–	–	–	–
*Rattus rattus*	Black rat	Rodentia	19	–	–	–	–	–
*Sciurus aestuans*	Guianan squirrel	Rodentia	1	–	–	–	–	–
*Felis wiedii*	Margay	Carnivora	1	–	–	–	1	–
*Puma yagouaroundi*	Jaguarundi	Carnivora	5	–	–	–	–	–
*Eira barbara*	Tayra	Carnivora	4	–	–	–	–	–
*Galictis vittata*	Greater grison	Carnivora	4	–	–	–	1	–
*Lontra longicaudis*	Neotropical river otter	Carnivora	1	–	–	–	–	–
*Potos flavus*	Kinkajou	Carnivora	2	–	–	–	–	–
*Alouatta macconnelli*	Guyanan red howler	Primates	22	–	–	–	–	–
*Saguinus midas*	Golden-handed tamarin	Primates	41	–	–	–	–	–
*Cebus apella*	Tufted capuchin	Primates	1	–	–	–	–	–
*Saimiri sciureus*	Guianan squirrel monkey	Primates	1	–	–	–	–	–
*Pithecia pithecia*	White-faced saki	Primates	1	–	–	–	–	–
**Birds**								
*Glyphorynchus spirurus*	Wedge-billed woodcreeper	Passeriformes	96	–	–	–	–	–
*Pipra aureola*	Crimson-hooded manakin	Passeriformes	36	–	–	–	–	–
*Ceratopipra erythrocephala*	Golden-headed manakin	Passeriformes	35	–	–	–	–	–
*Chiroxiphia pareola*	Blue-backed manakin	Passeriformes	44	–	–	–	–	–
*Myrmotherula axillaris*	White-flanked antwren	Passeriformes	36	–	–	–	–	–
**Ticks**								
*Ornithodoros capensis*	Seabird tick	Ixodida	6	–	–	–	–	–
*Amblyomma cajennense*	Cayenne tick	Ixodida	15	14	–	–	–	–
*Amblyomma calcaratum*	–	Ixodida	1	–	–	–	–	–
*Amblyomma coelebs*	–	Ixodida	31	–	–	–	–	–
*Amblyomma dissimile*	Iguana tick	Ixodida	21	–	–	–	2	11
*Amblyomma geayi*	–	Ixodida	10	–	–	–	–	–
*Amblyomma goeldii*	–	Ixodida	5	–	–	–	–	–
*Amblyomma humerale*	–	Ixodida	10	–	–	–	–	–
*Amblyomma latepunctatum*	–	Ixodida	4	–	–	–	–	–
*Amblyomma longirostre*	–	Ixodida	23	–	–	–	–	–
*Amblyomma naponense*	–	Ixodida	5	–	–	–	–	–
*Amblyomma oblongoguttatum*	–	Ixodida	42	–	–	–	1	–
*Amblyomma pacae*	–	Ixodida	6	–	–	–	–	–
*Amblyomma romitii*	–	Ixodida	2	–	–	–	–	–
*Amblyomma rotundatum*	–	Ixodida	6	–	–	–	–	–
*Amblyomma scalpturatum*	–	Ixodida	9	–	–	–	–	–
*Amblyomma varium*	Sloth’s giant tick	Ixodida	7	–	–	–	–	–
*Rhipicephalus microplus*	Asian blue tick/Tropical cattle tick	Ixodida	10	–	–	–	–	–
*Rhipicephalus sanguineus*	Brown dog tick	Ixodida	6	–	–	–	–	–
*Dermacentor nitens*	Tropical horse tick	Ixodida	55	–	–	–	–	–
*Haemaphysalis juxtakochi*	–	Ixodida	8	–	–	–	–	–
*Ixodes luciae*	Opossum tick	Ixodida	6	–	–	–	1	–
		Total	1161	21	3	–	15	11

### Molecular detection and typing of tick-borne Apicomplexa

Each DNA template underwent individual testing using semi-nested polymerase chain reaction (PCR) targeting a fragment of the 18S rRNA (SSU) gene to detect *Babesia*, *Theileria*, *Cytauxzoon*, *Hemolivia*, and *Hepatozoon* (Table S2). Semi-nested PCR amplifications were performed as follows: the first PCR run with the external primers was performed in a 10 μL volume containing 10–50 ng of genomic DNA, 3 mM of each dNTP (Thermo Scientific, Waltham, MA, USA), 8 mM of MgCl2 (Roche Diagnostics), 3 μM of each primer, 1 μL of 10× PCR buffer (Roche Diagnostics), and 0.5 U of Taq DNA polymerase (Roche Diagnostics). A 1 μL aliquot of the PCR product from the first reaction was then used as a template for the second round of amplification. The second PCR was performed in a total volume of 25 μL and contained 8 mM of each dNTP (Thermo Scientific), 10 mM of MgCl2 (Thermo Scientific), 7.5 μM of each of the internal primers, 2.5 μL of 10×PCR buffer (Thermo Scientific), and 1.25 U of Taq DNA polymerase (Thermo Scientific). Positive (DNA templates of *Dermacentor marginatus* ticks collected in the South of France and confirmed positive for *Babesia bovis*) and negative (water) controls were included in each PCR assay. Following visualization via electrophoresis in 1.5% agarose gel, positive PCR products were sequenced by Eurofins. Sequence chromatograms were cleaned with Chromas Lite (http://www.technelysium.com.au/chromas_lite.html). New sequences obtained in this study are available in GenBank under accession numbers PP476856–PP476867.

### Molecular phylogenetic analyses

Phylogenetic analyses were based on alignments of the Apicomplexa 18S rRNA gene sequences using MAFFT (https://mafft.cbrc.jp) for Piroplasmida and Eucoccidiorida, respectively. Sequences of Apicomplexa obtained from GenBank, including representative species of the genera *Babesia*, *Theileria*, *Cytauxzoon*, *Hemolivia*, and *Hepatozoon* were also included in the phylogenetic analyses. The Basic Local Alignment Search Tool (BLAST; https://blast.ncbi.nlm.nih.gov/blast/Blast.cgi) was used to find 18S rRNA gene sequences available on GenBank and showing the highest nucleotide similarities with the gene sequences we characterized in this study. The Gblocks program with default parameters was used to obtain non-ambiguous sequence alignments [16]. Phylogenetic analyses were performed using maximum-likelihood (ML) analyses on both alignments using the MEGA software package (https://www.megasoftware.net/). The evolutionary models that best fit the sequence data were determined using the Akaike information criterion. Clade robustness was assessed by bootstrap analysis using 1000 replicates.

## Results

### Detection of tick-borne Apicomplexa

Concerning the 71 species examined, molecular survey led to the identification of tick-borne Apicomplexa in 11 species (Tables 1 and S1). They were detected in seven species of mammals, including the common opossum (*Didelphis marsupialis*), gray four-eyed opossum (*Philander opossum*), Linnaeus’s two-toed sloth (*Choloepus didactylus*), nine-banded armadillo (*Dasypus novemcinctus*), porcupine (*Coendou* sp.), margay (*Felis wiedii*), and greater grison (*Galictis vittata*). They were also detected in four tick species, including the two species that most commonly bite humans in South America, the Cayenne tick (*Amblyomma cajennense*) and *Amblyomma oblongoguttatum*. The other two infected species were the opossum tick (*Ixodes luciae*), and the iguana tick (*Amblyomma dissimile*). No parasites were detected in the five species of passerines surveyed.

Among the 1161 specimens examined, we detected 50 positive samples (4.31%). On the basis of 18S rDNA gene sequences (452 bp), *Babesia* spp. were found in 21 samples (1.81%), *Theileria* sp. in three samples (0.26%), *Hemolivia* spp. in 11 samples (0.95%), and *Hepatozoon* spp. in 15 samples (1.29%) (Tables 1 and S1). No *Cytauxzoon* was detected. Specifically, *Babesia* spp. were present in seven Linnaeus’s two-toed sloths and 14 ticks (*A. cajennense*), *Theileria* spp. in three nine-banded armadillos, *Hemolivia* spp. in 11 ticks (*A. dissimile*), and *Hepatozoon* spp. in seven common opossums, one gray four-eyed opossum, one margay, one greater grison, and four ticks (one *I. luciae*, two *A. dissimile*, and one *A. oblongoguttatum*) (Tables 1 and S1). Infection prevalence was particularly high in *A. cajennense* (14 infected out of 15 examined, 99.33%), and *A. dissimile* (13 infected out of 21 examined, 61.90%). Infection prevalence was typically lower in mammals such as Linnaeus’s two-toed sloths (seven infected out of 90, 7.78%) and common opossums (seven infected out of 51, 13.73%).

### Molecular typing of tick-borne Apicomplexa

Sequencing of an 18S rDNA gene fragment from the 50 infected samples revealed the presence of eight Apicomplexa genovariants (Table S3). For piroplasmids, there were two genovariants of *Babesia* spp. (91.73% nucleotide identity between these two genovariants), and one genovariant of *Theileria* sp. (85.78%–87.97% nucleotide identities with *Babesia* genovariants). For hemogregarines, there were four genovariants of *Hepatozoon* spp. (96.31%–98.05% pairwise nucleotide identities), and one genovariant of *Hemolivia* sp. (96.10%–97.40% pairwise nucleotide identities with *Hepatozoon* genovariants). The *Hepatozoon* genovariant #2 was detected in opossums, porcupines, and the opossum tick *I. luciae*, suggesting a transmission cycle between these species in French Guiana. Similarly, the *Hepatozoon* genovariant #3 was present in margay and *A. oblongoguttatum*, a tick species feeding on a wide range of mammals. The other six genovariants were each found associated with only one species, either mammals or ticks (Table S3).

Three of the eight genovariants showed 100% nucleotide identity for their 18S rDNA sequences with known protozoan species (Table S3). *Hemolivia* genovariant #1, identified in *A. dissimile*, is identical to *Hemolivia stellata*, a parasite of the cane toad *Rhinella marina* [37]. *Hepatozoon* genovariant #3, detected in margay and *A. oblongoguttatum*, matched to *Hepatozoon luiperdjie*, a recently described parasite of the African leopard [6]. *Hepatozoon* genovariant #5, found in *A. dissimile*, matched to *Hepatozoon cuorae*, a parasite of Asian reptiles [43]. While *Hemolivia stellata* is native to South America [37], neither *Hepatozoon luiperdjie* nor *Hepatozoon cuorae* have been previously observed in the Americas [43]. The five other genovariants are distinct from taxa referenced in public databases based on their 18S rDNA sequences (Table S3).

### Phylogeny of tick-borne Apicomplexa

**Figure 1:**
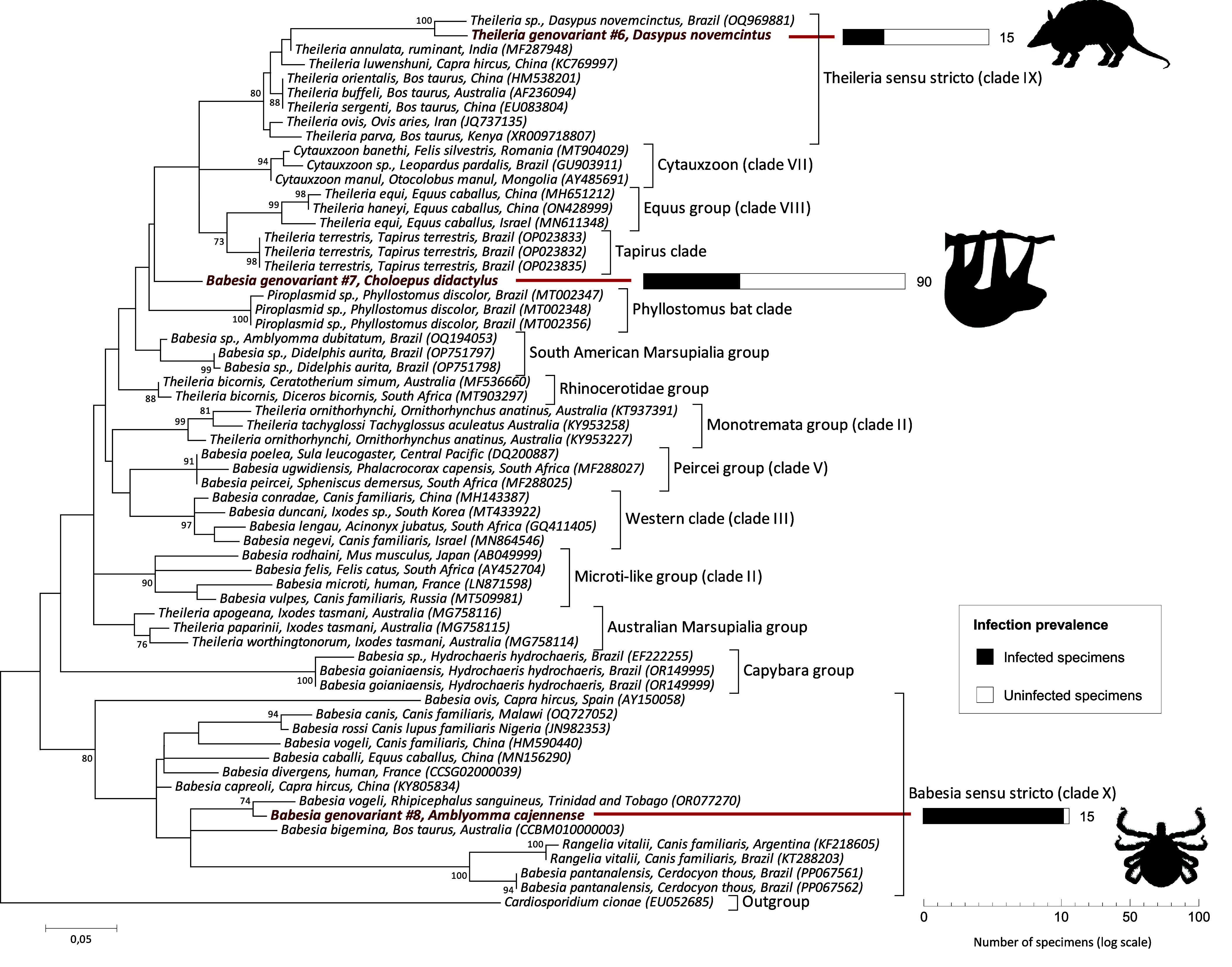
Phylogeny of piroplasmids (Piroplasmida order) constructed using maximum-likelihood (ML) estimations and based on 18S rDNA sequences (408 unambiguously aligned bp; best-fit approximation for the evolutionary model on the basis of Akaike information criterion: TN93+G+I). Only one 18S rDNA sequence per genovariant and per host species is shown for data produced in this study (in bold). GenBank accession numbers of sequences used in analyses are shown on the phylogenetic trees. Numbers at nodes indicate bootstrap support percentage with 1000 replicates. Only bootstrap values >70% are shown. The scale bar is in units of mean number of substitutions/site. The right part of the figure presents the prevalence of Apicomplexa infection observed for each infected species (on a logarithmic scale).

**Figure 2:**
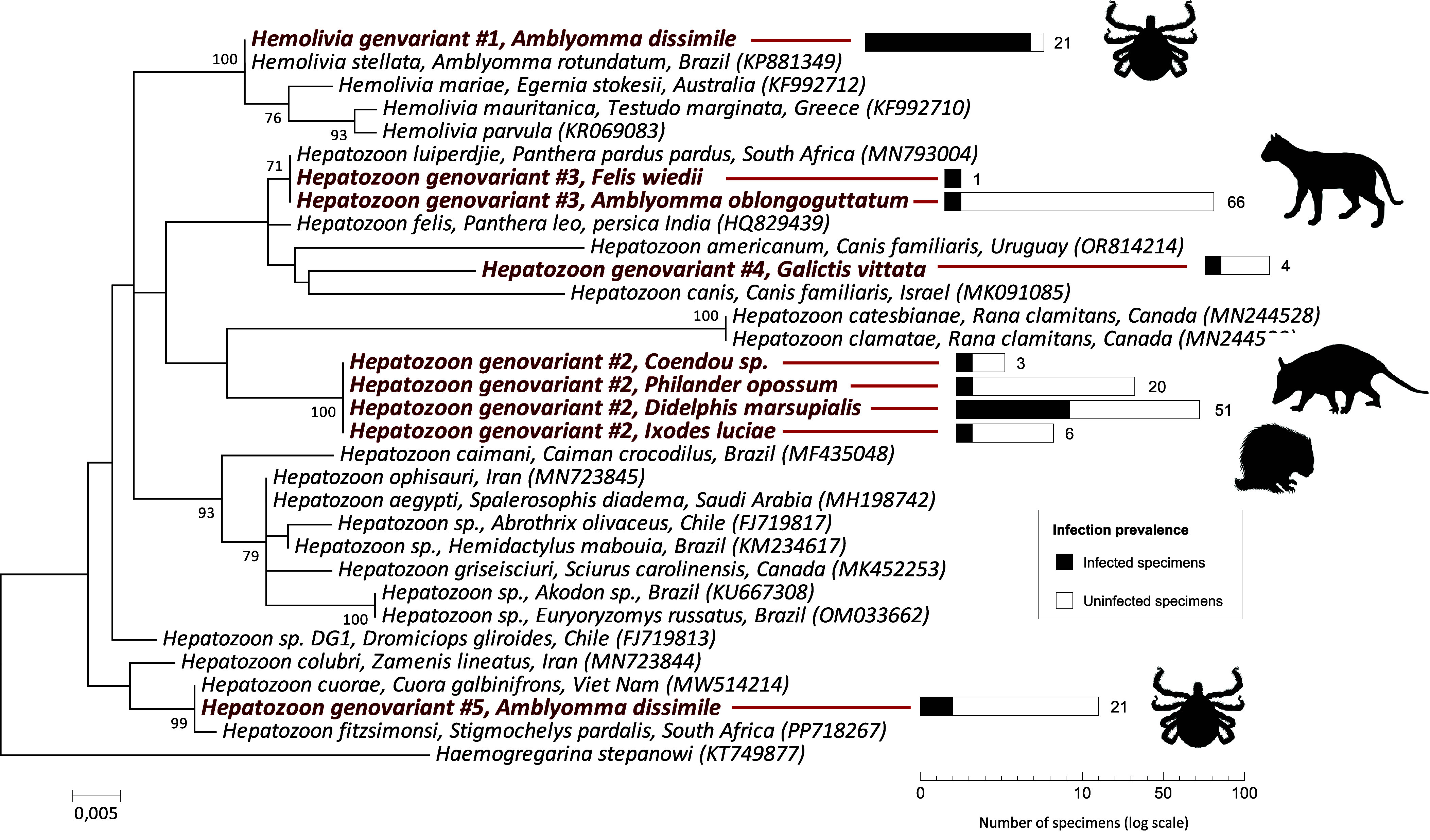
Phylogeny of tick-borne hemogregarines (Eucoccidiorida order) constructed using maximum-likelihood (ML) estimations based on 18S rDNA sequences (461 unambiguously aligned bp; best-fit approximation for the evolutionary model on the basis of Akaike information criterion: HKY+G). Only one 18S rDNA sequence per genovariant and per host species is shown for data produced in this study (in bold). GenBank accession numbers of sequences used in analyses are shown on the phylogenetic trees. Numbers at nodes indicate bootstrap support percentage with 1000 replicates. Only bootstrap values >70% are shown. The scale bar is in units of mean number of substitutions/site. The right part of the figure presents the prevalence of Apicomplexa infection observed for each infected species (on a logarithmic scale).

ML phylogenetic analyses based on 18S rRNA nucleotide sequences were further conducted to examine the phylogenetic proximity of tick-borne Apicomplexa detected in this study with representative species of the genera *Babesia*, *Theileria*, *Cytauxzoon*, *Hemolivia*, and *Hepatozoon*. ML analyses confirmed that (i) the two genovariants of *Babesia* (#7 and 8) and the genovariant of *Theileria* (#6) cluster with other piroplasmids (Fig. 1), (ii) the four genovariants of *Hepatozoon* (#2, 3, 4, and 5) and the genovariant of *Hemolivia* (#1) cluster with other *Hepatozoon* and *Hemolivia* species (Fig. 2). As expected [23], Piroplasmida is formed by the genus *Cytauxzoon* and a polyphyletic assemblage of *Babesia* and *Theileria* species.

ML analyses further showed that the *Babesia* genovariants #7 and #8 and the *Theileria* genovariant #6 are distantly related within the Order Piroplasmida (Fig. 1). *Babesia* genovariant #7 from Linnaeus’s two-toed sloths does not belong to a previously identified piroplasmid clade, including the recently described clades found in wildlife in Brazil, as the *Phyllostomus* bat clade [21], the *Tapirus* clade [30], the South American Marsupialia Group clade [31] and the Capybara clade [26]. *Babesia* genovariant #8 from *A. cajennense* belongs to the lineage *Babesia* sensu stricto (clade X in [23]), and is related to *Babesia vogeli*, one of the most important pathogens among *Babesia* species found in dogs [33]. *Theileria* genovariant #6 from nine-banded armadillos belongs to the *Theileria* sensu stricto lineage (clade IX in [23]), which include causative agents of tropical theileriosis, such as *Theileria orientalis* and *Theileria annulata*, two parasite species usually found in cattle [1]. In the *Theileria* sensu stricto lineage, *Theileria* genovariant #6 is closely related to another undescribed *Theileria* species recently found in nine-banded armadillos in Brazil (Fig. 1) [15, 39].

For hemogregarines, ML analyses confirmed the relatedness of *Hemolivia* genovariant #1 with *H. stellata*, and further indicated that they are both related to *Hemolivia parvula*, *Hemolivia mauritanica*, and *Hemolivia mariae* (Fig. 2), infecting African, Palearctic, and Australian reptiles, respectively [28, 50]. *Hepatozoon* genovariant #2 found in opossums, porcupines and the opossum tick *I. luciae* forms a branch that is well divergent from all other *Hepatozoon* species, with no close relatedness to known species or isolates. *Hepatozoon* genovariant #3 clusters with *Hepatozoon luiperdjie*, and both are closely related to the common cat parasite *Hepatozoon felis*. The *Hepatozoon* genovariant #4 we detected in greater grison is more closely related to *Hepatozoon canis* and *Hepatozoon americanum*, which infect dogs in the Americas. *Hepatozoon* genovariant #5 clusters with *Hepatozoon cuorae* and *Hepatozoon colubri*, which both infect turtles and snakes (Fig. 2) [44, 48, 49].

## Discussion

In our study, we have identified several genovariants of *Babesia*, *Theileria*, *Hemolivia*, and *Hepatozoon* in various wildlife species, including opossums, sloths, armadillos, and porcupines, as well as in tick species that most commonly bite humans in South America. While some genovariants match with known species, others do not, raising evidence of undescribed Apicomplexa species in French Guiana. The detection of similar genovariants in wildlife and ticks further unveils the existence of unique sylvatic transmission cycles for these Apicomplexa. These observations in French Guiana remarkably mirror recent surveys of wildlife in Brazil, which also highlight the distinctiveness of South American tick-borne Apicomplexa and their specific sylvatic transmission cycles [4, 5, 13, 20, 21, 26, 30, 31, 34, 36, 43].

None of the three piroplasmid genovariants found in French Guiana matched with *Babesia*, *Theileria*, and *Cytauxzoon* species, strains or isolates for which 18S rRNA sequences were available in public databases. They may each represent a new putative species or an already described species for which no molecular sequences are currently available. Indeed, *Babesia choloepi*, which was morphologically characterized from erythrocytes of Linnaeus’s two-toed sloths of French Guiana [18], may be the *Babesia* genovariant #7 we characterized also in Linnaeus’s two-toed sloths. This *Babesia* genovariant is distinct from all already known piroplasmid clades, including those recently described in wildlife in Brazil [21, 26, 30, 31], suggesting it may form a novel piroplasmid clade. The *Theileria* genovariant #6 of the nine-banded armadillo is similar to a *Theileria* sp. infected in nine-banded armadillos in Brazil [15, 39], and both may be related to another *Theileria* sp., observed through microscopy in nine-banded armadillos in the state of Pará, Brazil, as mentioned by Lainson et al*.* [29], but not further described. The association of armadillos with the *Theileria* genovariant #6 is more singular since this parasite is related to species of the *Theileria* sensu stricto lineage and that are usually found in cattle [23]. The *Babesia* genovariant #8 is closely related to *Babesia vogeli*, which is commonly found in dogs and cats in Brazil [4]. Its detection in *A. cajennense*, a tick species feeding on a wide range of mammals, including humans, suggests that a diverse range of hosts is exposed to this parasite. However, *Babesia* genovariant #8 has never been detected in vertebrates, indicating that its natural hosts are currently unknown.

The diversity of *Hemolivia* and *Hepatozoon* genovariants found in French Guiana leads to quite similar observations. *Hemolivia* genovariant #1 is evidently *Hemolivia stellata*, a parasite that colonizes erythrocytes, cells of the reticuloendothelial system, and digestive cells of the cane toad [37]. However, while *A. rotundatum* is vector of this parasite in Brazil [37], we commonly observed infection in another tick species in French Guiana, the iguana tick *A. dissimile.* This tick species specializes in reptiles and amphibians, commonly feeding on cane toads, and may thus serve as an additional vector species of *Hemolivia stellata* in French Guiana. Similarly, *Hepatozoon* genovariant #5 is related to *Hepatozoon cuorae*, a species known to infect Asiatic turtles [49]. However, *Hepatozoon cuorae* has never been observed in South America, but our observation suggests that a related species may exist in French Guiana. *Amblyomma dissimile* is a potential local vector, and given the host specificity of this tick species, this parasite may infect South American reptiles or amphibians or both. *Hepatozoon* genovariant #3 is related to *Hepatozoon luiperdjie*, a parasite of the African leopard [6], previously not found outside Africa. The detection of this genovariant in margays of French Guiana, and in *A. oblongoguttatum* as a potential local vector, suggests that the *Hepatozoon luiperdjie* relatives may form a clade specific to wild felids across different continents. *Hepatozoon* genovariant #4 found in greater grison is different to all known species but is related to *Hepatozoon canis* and *Hepatozoon americanum*, forming together a large clade associated with carnivores. On the contrary, the discovery of *Hepatozoon* genovariant #2 in two opossum species, porcupines, and the opossum tick *I. luciae* indicates that this parasite is common and widespread in the rainforests of French Guiana. However, *Hepatozoon* genovariant #2 has not been documented elsewhere, prompting inquiries into its geographic distribution.

In conclusion, the investigation into tick-borne Apicomplexa in French Guiana underscores the significance of acknowledging the diversity of these infections within this region. Moreover, the identification of potential novel species emphasizes the urgent need for ongoing research and surveillance to mitigate the health risks posed by these tick-borne parasites. A major limitation of our study is that we used a short fragment of the 18S rDNA for the typing of infection. Complete sequencing of the 18S rDNA and additional markers, such as mitochondrial markers, are now needed to confirm the existence of new species and new clades. Notably, these new typings should allow for better comparison with the new species discovered in South America, particularly for *Hepatozoon*, for which many new genovariants have been described [2, 3, 13, 35, 40].

## Supplementary material

The supplementary material for this article can be found at https://www.parasite-journal.org/10.1051/parasite/2024052/olm. *Table S1:* List and locations of samples examined for the presence of tick-borne Apicomplexa in French Guiana. *Table S2:* Primers used in polymerase chain reaction (PCR) assays and Sanger sequencing for 18S rDNA (SSU) gene of tick-borne Apicomplexa. *Table S3:* Genovariants of *Babesia*, *Theileria*, *Hemolivia* and *Hepatozoon* detected in French Guiana and best nucleotide identities with 18S rRNA gene sequences obtained in this study and sequences available in GenBank identified through Basic Local Alignment Search Tool (BLAST; https://blast.ncbi.nlm.nih.gov/Blast.cgi) searches (Last update: 01/04/2024).

## References

[1] Agina OA, Shaari MR, Isa NMM, Ajat M, Zamri-Saad M, Hamzah H. 2020. Clinical pathology, immunopathology and advanced vaccine technology in bovine theileriosis: a review. Pathogens, 9, 1–22. 10.3390/pathogens9090697 PMC7558346 32854179

[2] Alabí AS, Monti G, Otth C, Sepulveda-García P, Perles L, Machado RZ, André MR, Bittencourt P, Müller A. 2021. Genetic diversity of *Hepatozoon* spp. in rodents from Chile. Revista Brasileira de Parasitologia Veterinaria, 30, e012721. 34755807 10.1590/S1984-29612021082

[3] André MR, Adania CH, Teixeira RHF, Vargas GH, Falcade M, Sousa L, Salles AR, Allegretti SM, Felippe PAN, Machado RZ. 2010. Molecular detection of *Hepatozoon* spp. in Brazilian and exotic wild carnivores. Veterinary Parasitology, 173, 134–138. 20630658 10.1016/j.vetpar.2010.06.014

[4] André MR, Calchi AC, Furquim MEC, de Andrade I, Arantes PVC, de Lopes LCM, Demarchi IKLDN, Figueiredo MAP, de Paula Lima CA, Machado RZ. 2022. Molecular detection of tick-borne agents in cats from Southeastern and Northern Brazil. Pathogens, 11, 106. 35056054 10.3390/pathogens11010106 PMC8781600

[5] André MR, Calchi AC, Perles L, Gonçalves LR, Uccella L, Lemes JRB, Nantes WAG, Santos FM, de Oliveira Porfírio GE, Barros-Battesti DM, Herrera HM, Machado RZ. 2022. Novel *Ehrlichia* and *Hepatozoon* genotypes in white-eared opossums (*Didelphis albiventris*) and associated ticks from Brazil. Ticks and Tick-Borne Diseases, 13, 102022. 35973262 10.1016/j.ttbdis.2022.102022

[6] Van As M, Netherlands EC, Smit NJ. 2020. Molecular characterisation and morphological description of two new species of *Hepatozoon* Miller, 1908 (Apicomplexa: Adeleorina: Hepatozoidae) infecting leukocytes of African leopards *Panthera pardus pardus* (L.). Parasites & Vectors, 13, 222. 32357916 10.1186/s13071-020-3933-6 PMC7195708

[7] Binetruy F, Buysse M, Barosi R, Duron O. 2020. Novel *Rickettsia* genotypes in ticks in French Guiana, South America. Scientific Reports, 10, 10–14. 32054909 10.1038/s41598-020-59488-0 PMC7018960

[8] Binetruy F, Buysse M, Lejarre Q, Barosi R, Villa M, Rahola N, Paupy C, Ayala D, Duron O. 2020. Microbial community structure reveals instability of nutritional symbiosis during the evolutionary radiation of *Amblyomma* ticks. Molecular Ecology, 29, 1016–1029. 32034827 10.1111/mec.15373

[9] Binetruy F, Chevillon C, de Thoisy B, Garnier S, Duron O. 2019. Survey of ticks in French Guiana. Ticks and Tick-Borne Diseases, 10, 77–85. 30224310 10.1016/j.ttbdis.2018.09.003

[10] Binetruy F, Dupraz M, Buysse M, Duron O. 2019. Surface sterilization methods impact measures of internal microbial diversity in ticks. Parasites & Vectors, 12, 268. 31138324 10.1186/s13071-019-3517-5 PMC6537145

[11] Binetruy F, Duron O. 2023. Molecular detection of *Cercopithifilaria*, *Cruorifilaria* and *Dipetalonema*-like filarial nematodes in ticks of French Guiana. Parasite, 30, 24. 37404115 10.1051/parasite/2023027 PMC10321233

[12] Binetruy F, Garnier S, Boulanger N, Talagrand-Reboul É, Loire E, Faivre B, Noël V, Buysse M, Duron O. 2020. A novel *Borrelia* species, intermediate between Lyme disease and relapsing fever groups, in neotropical passerine-associated ticks. Scientific Reports, 10, 10596. 32606328 10.1038/s41598-020-66828-7 PMC7327063

[13] Braga MD, Costa FB, Calchi AC, de Mello VV, Mongruel AC, Dias CM, Bassini-Silva R, Silva EM, Pereira JG, dos Santos Ribeiro LS, da Costa AP, de Andrade FHE, Silva ALA, Machado RZ, André MR. 2023. Molecular detection and characterization of vector-borne agents in common opossums (*Didelphis marsupialis*) from northeastern Brazil. Acta Tropica, 244, 106955. 37236334 10.1016/j.actatropica.2023.106955

[14] Buysse M, Koual R, Binetruy F, de Thoisy B, Baudrimont X, Garnier S, Douine M, Chevillon C, Delsuc F, Catzeflis F, Bouchon D, Duron O. 2024. Detection of *Anaplasma* and *Ehrlichia* bacteria in humans, wildlife, and ticks in the Amazon rainforest. Nature Communications, 15, 3988. 10.1038/s41467-024-48459-y PMC11088697 38734682

[15] Calchi AC, Yogui DR, Alves MH, Desbiez ALJ, Kluyber D, Vultão JG, Arantes PVC, de Santi M, Werther K, Teixeira MMG, Machado RZ, André MR. 2023. Molecular detection of piroplasmids in mammals from the Superorder Xenarthra in Brazil. Parasitology Research, 122, 3169–3180. 37848747 10.1007/s00436-023-08008-w

[16] Castresana J. 2000. Selection of conserved blocks from multiple alignments for their use in phylogenetic analysis. Molecular Biology and Evolution, 17, 540–552. 10742046 10.1093/oxfordjournals.molbev.a026334

[17] Chauvin A, Moreau E, Bonnet S, Plantard O, Malandrin L. 2009. *Babesia* and its hosts: adaptation to long-lasting interactions as a way to achieve efficient transmission. Veterinary Research, 40, 37. 19379662 10.1051/vetres/2009020 PMC2695028

[18] Dedet JP, Veilly M, Robin Y, Bonnevie O, Landau I. 1988. *Babesia choloepi* n. sp. (Apicomplexa, Piroplasmida), parasite du paresseux à deux doigts, *Choloepus didactylus* (Linné, 1758) (Xenarthra, Bradypodidae), en Guyane française. Annales de Parasitologie Humaine et Comparée, 63, 16–21. 3400959 10.1051/parasite/198863116

[19] Duron O, Koual R, Musset L, Buysse M, Lambert Y, Jaulhac B, Blanchet D, Alsibai KD, Lazrek Y, Epelboin L, Deshuillers P, Michaud C, Douine M. 2022. Novel chronic anaplasmosis in splenectomized patient, Amazon rainforest. Emerging Infectious Diseases, 28, 1673–1676. 35876693 10.3201/eid2808.212425 PMC9328922

[20] Gonçalves LR, Paludo G, Bisol TB, Perles L, de Oliveira LB, de Oliveira CM, da Silva TMV, Nantes WAG, Duarte MA, Santos FM, de Oliveira Porfírio GE, Hirano LQL, Herrera HM, Barros-Battesti DM, Machado RZ, André MR. 2021. Molecular detection of piroplasmids in synanthropic rodents, marsupials, and associated ticks from Brazil, with phylogenetic inference of a putative novel *Babesia* sp. from white-eared opossum (*Didelphis albiventris*). Parasitology Research, 120, 3537–3546. 34448058 10.1007/s00436-021-07284-8

[21] Ikeda P, Menezes TR, Torres JM, de Oliveira CE, Lourenço EC, Herrera HM, Machado RZ, André MR. 2021. First molecular detection of piroplasmids in non-hematophagous bats from Brazil, with evidence of putative novel species. Parasitology Research, 120, 301–310. 33244622 10.1007/s00436-020-06985-w

[22] Jalovecka M, Hajdusek O, Sojka D, Kopacek P, Malandrin L. 2018. The complexity of piroplasms life cycles. Frontiers in Cellular and Infection Microbiology, 8, 248. 30083518 10.3389/fcimb.2018.00248 PMC6065256

[23] Jalovecka M, Sojka D, Ascencio M, Schnittger L. 2019. *Babesia* life cycle – When phylogeny meets biology. Trends in Parasitology, 35, 356–368. 30733093 10.1016/j.pt.2019.01.007

[24] Kjemtrup AM, Thomford J, Robinson T, Conrad PA. 2000. Phylogenetic relationships of human and wildlife piroplasm isolates in the western United States inferred from the 18S nuclear small subunit RNA gene. Parasitology, 120(Pt 5), 487–493. 10840978 10.1017/s003118209900582x

[25] Koual R, Buysse M, Grillet J, Binetruy F, Ouass S, Sprong H, Duhayon M, Boulanger N, Jourdain F, Alafaci A, Verdon J, Verheyden H, Rispe C, Plantard O, Duron O. 2023. Phylogenetic evidence for a clade of tick-associated trypanosomes. Parasites & Vectors, 16, 3. 36604731 10.1186/s13071-022-05622-y PMC9817367

[26] Krawczak FDS, Calchi AC, Neves LC, Dias SA, da Silva BBF, Paula WVDF, de Paula LGF, Tavares MA, Pádua GT, de Lima NJ, Cardoso ERN, Graziani D, Dantas-Torres F, André MR. 2023. Phylogenetic inferences based on distinct molecular markers confirm a novel *Babesia* species (*Babesia goianiaensis* nov. sp.) in capybaras (*Hydrochoerus hydrochaeris*) and associated ticks. Microorganisms, 11, 2022. 37630582 10.3390/microorganisms11082022 PMC10459827

[27] Kukina IV, Guzeeva TM, Zelya OP, Ganushkina LA. 2018. Fatal human babesiosis caused by *Babesia divergens* in an asplenic host. IDCases, 13, 4. 10.1016/j.idcr.2018.e00414 PMC6070674 30073148

[28] Kvičerová J, Hypša V, Dvořáková N, Mikulíček P, Jandzik D, Gardner MG, Javanbakht H, Tiar G, Široký P. 2014. *Hemolivia* and *Hepatozoon*: haemogregarines with tangled evolutionary relationships. Protist, 165, 688–700. 25233121 10.1016/j.protis.2014.06.001

[29] Lainson R, Shaw JJ, Fraiha H, Miles MA, Draper CC. 1979. Chagas’s Disease in the Amazon Basin: 1. *Trypanosoma cruzi* infections in silvatic mammals, triatomine bugs and man in the State of Pará, north Brazil. Transactions of the Royal Society of Tropical Medicine and Hygiene, 73, 193–204. 112730 10.1016/0035-9203(79)90211-6

[30] Mongruel ACB, Medici EP, da Costa Canena A, Calchi AC, Perles L, Rodrigues BCB, Soares JF, Machado RZ, André MR. 2022. *Theileria terrestris* nov. sp.: a novel *Theileria* in lowland tapirs (*Tapirus terrestris*) from two different biomes in Brazil. Microorganisms, 10, 2319. 36557572 10.3390/microorganisms10122319 PMC9784709

[31] de Oliveira ÁFX, Calchi AC, Stocco AV, Stocco NV, Costa AC, Mureb EN, Pires JR, Guimarães A, Raimundo JM, de Almeida Balthazar D, Machado RZ, André MR, Baldani CD. 2023. Expanding the universe of Piroplasmids: morphological detection and phylogenetic positioning of putative novel piroplasmids in black-eared opossums (*Didelphis aurita*) from southeastern Brazil, with description of “South American Marsupialia Group” of Piroplasmida. Parasitology Research, 122, 1519–1530. 37195507 10.1007/s00436-023-07852-0

[32] Ouass S, Boulanger N, Lelouvier B, Insonere J-L-M, Lacroux C, Krief S, Asalu E, Rahola N, Duron O. 2023. Diversity and phylogeny of the tick-borne bacterial genus *Candidatus* Allocryptoplasma (Anaplasmataceae). Parasite, 30, 13. 37162293 10.1051/parasite/2023014 PMC10171070

[33] Penzhorn BL. 2020. Don’t let sleeping dogs lie: unravelling the identity and taxonomy of *Babesia canis*, *Babesia rossi* and *Babesia vogeli*. Parasites & Vectors, 13, 184. 32312292 10.1186/s13071-020-04062-w PMC7171786

[34] Perles L, Barreto WTG, de Macedo GC, Calchi AC, Bezerra-Santos M, Mendoza-Roldan JA, Otranto D, Herrera HM, Barros-Battesti DM, Machado RZ, André MR. 2023. Molecular detection of *Babesia* spp. and *Rickettsia* spp. in coatis (*Nasua nasua*) and associated ticks from midwestern Brazil. Parasitology Research, 122, 1151–1158. 36890298 10.1007/s00436-023-07815-5

[35] Perles L, Roque ALR, D’Andrea PS, Lemos ERS, Santos AF, Morales AC, Machado RZ, André MR. 2019. Genetic diversity of *Hepatozoon* spp. in rodents from Brazil. Scientific Reports, 9, 1–9. 10.1038/s41598-019-46662-2 PMC6626033 31300712

[36] Perles L, Ikeda P, de Vasconcellos Francisco G, Torres JM, de Oliveira CE, Lourenço EC, Herrera HM, Machado RZ, André MR. 2020. Molecular detection of *Hepatozoon* spp. in non-hematophagous bats in Brazil. Ticks and Tick-Borne Diseases, 11, 101401. 32014465 10.1016/j.ttbdis.2020.101401

[37] Petit G, Landau I, Baccam D, Lainson R. 1990. Description et cycle biologique d’*Hemolivia stellata* n. g., n. sp., hémogrégarine de crapauds brésiliens. Annales de Parasitologie Humaine et Comparée, 65, 3–15.

[38] Smith TG. 1996. The genus *Hepatozoon* (Apicomplexa: Adeleina). Journal of Parasitology, 82, 565–585. 8691364

[39] Soares HS, Marcili A, Barbieri ARM, Minervino AHH, Moreira TR, Gennari SM, Labruna MB. 2017. Novel piroplasmid and *Hepatozoon* organisms infecting the wildlife of two regions of the Brazilian Amazon. International Journal for Parasitology: Parasites and Wildlife, 6, 115–121. 28603688 10.1016/j.ijppaw.2017.05.002 PMC5454132

[40] de Sousa KCM, Fernandes MP, Herrera HM, Benevenute JL, Santos FM, Rocha FL, Barreto WTG, Macedo GC, Campos JB, Martins TF, de Andrade Pinto PCE, Battesti DB, Piranda EM, Cançado PHD, Machado RZ, André MR. 2017. Molecular detection of *Hepatozoon* spp. in domestic dogs and wild mammals in southern Pantanal, Brazil with implications in the transmission route. Veterinary Parasitology, 237, 37–46. 28291601 10.1016/j.vetpar.2017.02.023

[41] de Thoisy B, Michel JC, Vogel I, Vié JC. 2000. A survey of hemoparasite infections in free-ranging mammals and reptiles in French Guiana. Journal of Parasitology, 86, 1035–1040. 11128476 10.1645/0022-3395(2000)086[1035:ASOHII]2.0.CO;2

[42] de Thoisy B, Duron O, Epelboin L, Musset L, Quénel P, Roche B, Binetruy F, Briolant S, Carvalho L, Chavy A, Couppié P, Demar M, Douine M, Dusfour I, Epelboin Y, Flamand C, Franc A, Ginouvès M, Gourbière S, Houël E, Kocher A, Lavergne A, Le Turnier P, Mathieu L, Murienne J, Nacher M, Pelleau S, Prévot G, Rousset D, Roux E, Schaub R, Talaga S, Thill P, Tirera S, Guégan JF. 2021. Ecology, evolution, and epidemiology of zoonotic and vector-borne infectious diseases in French Guiana: transdisciplinarity does matter to tackle new emerging threats. Infection Genetics and Evolution, 93, 104916. 10.1016/j.meegid.2021.104916 34004361

[43] Thomas R, Santodomingo A, Saboya-Acosta L, Quintero-Galvis JF, Moreno L, Uribe JE, Muñoz-Leal S. 2024. *Hepatozoon* (Eucoccidiorida: Hepatozoidae) in wild mammals of the Americas: a systematic review. Parasites & Vectors, 17, 108. 38444020 10.1186/s13071-024-06154-3 PMC10916324

[44] Tila H, Khan M, Almutairi MM, Alouffi A, Ahmed H, Tanaka T, Tsai KH, Ali A. 2023. First report on detection of *Hepatozoon ayorgbor* in *Rhipicephalus haemaphysaloides* and *Hepatozoon colubri* in *Haemaphysalis sulcata* and *Hyalomma anatolicum*: risks of spillover of *Hepatozoon* spp. from wildlife to domestic animals. Frontiers in Veterinary Science, 10, 1255482. 37789871 10.3389/fvets.2023.1255482 PMC10544907

[45] Vannier EG, Diuk-Wasser MA, Ben Mamoun C, Krause PJ. 2015. Babesiosis. Infectious Disease Clinics of North America, 29, 357–370. 25999229 10.1016/j.idc.2015.02.008 PMC4458703

[46] Westblade LF, Simon MS, Mathison BA, Kirkman LA. 2017. *Babesia microti*: from mice to ticks to an increasing number of highly susceptible humans. Journal of Clinical Microbiology, 55, 2903–2912. 28747374 10.1128/JCM.00504-17 PMC5625376

[47] Yabsley MJ, Shock BC. 2012. Natural history of zoonotic *Babesia*: Role of wildlife reservoirs. International Journal for Parasitology: Parasites and Wildlife, 2, 18–31. 24533312 10.1016/j.ijppaw.2012.11.003 PMC3862492

[48] Zechmeisterová K, Javanbakht H, Kvičerová J, Široký P. 2021. Against growing synonymy: identification pitfalls of *Hepatozoon* and *Schellackia* demonstrated on north Iranian reptiles. European Journal of Protistology, 79, 125780. 34020115 10.1016/j.ejop.2021.125780

[49] Zechmeisterová K, Přibyl M, Manh Nguyen H, Nosková E, Široký P. 2022. Haemogregarines of the genera *Haemogregarina*, *Hemolivia*, and *Hepatozoon* infecting vietnamese freshwater turtles, with additional notes on primer specificity and primer-template mismatches affecting diagnostic success. Protist, 173, 125884. 35843169 10.1016/j.protis.2022.125884

[50] Živčicová Ž, Kvičerová J, Široký P. 2024. *Hemolivia* species infecting Central American wood turtles (*Rhinoclemmys pulcherrima manni*) and problems with differential diagnosis within the genus *Hemolivia*. Parasite, 31, 1. 38353582 10.1051/parasite/2023067 PMC10865994

