# Supplementary material for: Tick-borne Apicomplexa in wildlife and ticks of French Guiana
Source: Parasite. 2024 Aug 15;31:49. doi: 10.1051/parasite/2024052 (PMC11334698; doi:10.1051/parasite/2024052)
Supplement: Supplementary file 2 — Table S2: Primers used in polymerase chain reaction (PCR) assays and Sanger sequencing for 18S rDNA (SSU) gene of tick-borne Apicomplexa. [file parasite-31-49-s2.pdf]

Table S2. Primers used in polymerase chain reaction (PCR) assays and Sanger sequencing for 18S rDNA (SSU) gene of tick-borne Apicomplexa.

Gene	Product	Primers (5'-3')	Fragment size	Refere nce
18S rRNA	Eukaryotic ribosomal small subunit (SSU rRNA)	TBPP_18S_310_F1 - GCCTACCGRGGCARCAACG	1st round PCR: TBPP_18S_310_F1 / TBPP_18S_993_R: 706 bp	This study
		TBPP_18S_579_F2 - GCGTATATTAAASTTGTTGCAG	2nd round PCR: TBPP_18S_579_F1 / TBPP_18S_993_R: 452 bp	
		TBPP_18S_993_R - CTTCAGSASCTTGAGAGAAATC		
