# Supplementary material for: Tick-borne Apicomplexa in wildlife and ticks of French Guiana
Source: Parasite. 2024 Aug 15;31:49. doi: 10.1051/parasite/2024052 (PMC11334698; doi:10.1051/parasite/2024052)
Supplement: Supplementary file 3 — Table S3: Genovariants of Babesia, Theileria, Hemolivia and Hepatozoon detected in French Guiana and best nucleotide identities with 18S rRNA gene sequences obtained in this study and sequences available in GenBank identified through Basic Local Alignment Search Tool (BLAST; https://blast.ncbi.nlm.nih.gov/Blast.cgi) searches (Last update: 01/04/2024). [file parasite-31-49-s3.pdf]

Table S3. Genovariants of *Babesia*, *Theileria*, *Hemolivia* and *Hepatozoon* detected in French Guiana and best nucleotide identities with 18S rRNA gene sequences obtained in this study and sequences available in GenBank identified through Basic Local Alignment Search Tool (BLAST; https://blast.ncbi.nlm.nih.gov/Blast.cgi) searches (Last update: 01/04/2024).

Genovariant	Host species	Number of infected specimens	Best match in Genbank [% nucleotide identity; length of the 18S rRNA gene sequence; query cover; E-value; accession number]	Best match in the present study (% nucleotide identity)
Hemolivia#1	*Amblyomma dissimile* (iguana tick)	11	*Hemolivia stellata*, hosted by the tick *Amblyomma rotundatum*, Brazil [100%; 462 bp; 100%; E=0.0; KP881349]	*Hepatozoon* genovariant #3 of *Felis wiedii* (97.40%)
Hepatozoon #2	*Didelphis marsupialis* (common opossum)	7	*Hepatozoon* sp. DG1, hosted by the colocolo opossum *Dromiciops gliroides*, Chile [97.63%; 462 bp; 100%; E=0.0; FJ719813]	*Hepatozoon* genovariant #3 of *Felis wiedii* (98.05%)
	Philander opossum (gray four-eyed opossum)	1		
	Coendou sp. (prehensile-tailed porcupine)	1		
	Ixodes luciae (opossum tick)	1		
Hepatozoon #3	*Felis wiedii* (margay)	1	*Hepatozoon luiperdjie*, hosted by the African leopard *Panthera pardus pardus*, South Africa [100%; 462 bp; 100%; E=0.0; MN793004]	*Hepatozoon* genovariant #2 of opossums (98.05%)
	Amblyomma oblongoguttatum (tick)	1		
Hepatozoon #4	*Galictis vittata* (greater grison)	1	*Hepatozoon felis*, hosted by the domestic cat *Felis silvestris*, Hungary [98.05%; 462 bp; 100%; E=0.0; OM422756]	*Hepatozoon* genovariant #3 of *Felis wiedii* (97.83%)
Hepatozoon #5	*Amblyomma dissimile* (iguana tick)	2	*Hepatozoon cuorae*, hosted by the Indochinese box turtle *Cuora galbinifrons*, Viet Nam [100%; 462 bp; 100%; E=0.0; MW514214]	*Hepatozoon* genovariant #2 of opossums (97.41%)
Theileria #6	*Dasypus novemcinctus* (nine-banded armadillo)	3	*Theileria luwenshun*, hosted by domestic ruminants, China [90.41%; 462 bp; 100%; E=3e-155; KC769997]	*Babesia* genovariant #8 of *Amblyomma cajennense* (87.97%)
Babesia #7	*Choloepus didactylus* (two-toed sloth)	7	*Babesia* sp. isolate ID21ND, hosted by the tick *Amblyomma dubitatum*, Brazil [92.97%; 462 bp; 100%; E=1e-180; OQ194053]	*Babesia* genovariant #8 of *Amblyomma cajennense* (91.73%)
Babesia #8	*Amblyomma cajennense* (Cayenne tick)	14	*Babesia vogeli*, hosted by the brown dog tick *Rhipicephalus sanguineus*, Trinidad and Tobago [97.98%; 462 bp; 99%; E=1e-180; OR077270]	*Babesia* genovariant #7 of *Choloepus didactylus* (91.73%)
